# Supplementary material for: The Value of CXCL1, CXCL2, CXCL3, and CXCL8 as Potential Prognosis Markers in Cervical Cancer: Evidence of E6/E7 from HPV16 and 18 in Chemokines Regulation
Source: Biomedicines. 2023 Sep 28;11(10):2655. doi: 10.3390/biomedicines11102655 (PMC10604789; doi:10.3390/biomedicines11102655)
Supplement: Supplementary file 1 [file biomedicines-11-02655-s001.zip › biomedicines-2615396-supplementary.pdf]

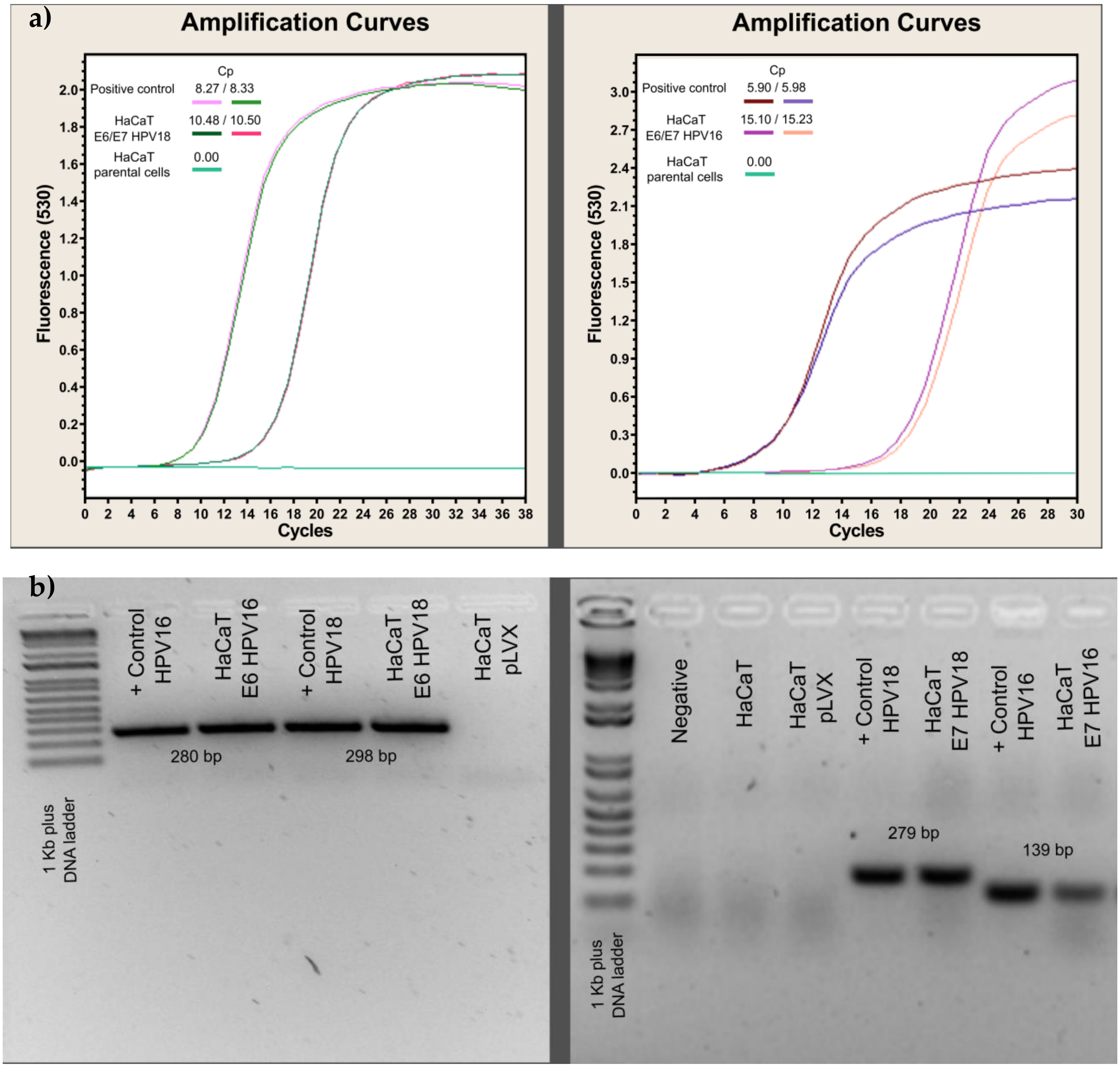


**c)** Primer sets sequences used for amplification of E6 and E7 oncogenes.

| Primer | Fwd. Sequence | Rev. Sequence | Product length |
|---|---|---|---|
| *E6* HPV16 | 5′ CAG AGC TGC AAA CAA CTA TAC 3′ | 5′ AGT GGC TTT TGA CAG TTA ATA C 3′ | 280 bp |
| *E6* HPV18 | 5′ GCG ACC CTA CAA GCT ACC TGA T 3′ | 5′ GCA CCG CAG GCA CCT TAT TA 3′ | 298 bp |
| *E7* HPV16 | 5′ GAC AAG CAG AAC CGG ACA G 3′ | 5′ ATT CCT AGT GTG CCC ATT AAC A 3′ | 139 bp |
| *E7* HPV18 | 5′ TGT CAC GAG CAA TTA AGC GAC T 3′ | 5′ CAC ACA AAG GAC AGG GTG TTC A 3′ | 279 bp |

**Figure S1. Validation of E6/E7 expression on HaCaT-transduced cell models. a)** The graphs shown the amplification curves and CPs derived from real-time-PCR utilizing specific primers for *E6* and *E7* oncogenes from HPV 16 and 18. Pure plasmid LVX with E6/E7 of HPV 16 and 18 were included as positive controls, and HaCaT parental cells were also included. **b)** Electrophoresis on 2% agarose gels were addressed to demonstrate the presence of the amplicon; we expected the following lengths: E6 HPV16 = 280 bp, E6 HPV18 = 298 bp, E7 HPV16 = 139 bp, and E7 HPV18 = 279 bp. Positive controls: Pure plasmids LVX with E6/E7 of HPV 16 or 18; Negative controls: HaCaT and HaCaT pLVX. **c)** Sequences used to amplify *E6* or *E7* from HPV16 or 18.

**Supplementary Table S1.** Primer sets sequences used in qPCR.

| Primer | Fwd. Sequence | Rev. Sequence | Product length | Annealing temp. |
|---|---|---|---|---|
| *CCL2* | 5´ GCA GCA AGT GTC CCA A 3´ | 5´CCC AAG TCT CTG TAT CTA AAA 3´ | 337 bp | 54°C |
| *CCL28* | 5´ AGC TGT TGC ACG GAG GTT T 3´ | 5´ TTC TTG GCA GCT TGC ACT TTC 3´ | 191bp | 60°C |
| *CXCL1* | 5´TCA AGA ATG GGC GGA AAG 3´ | 5´CTT CTC CTA AGC GAT GCT CAA 3´ | 231 bp | 58°C |
| *CXCL2* | 5´ GCT TGT CTC AAC CCC GCA 3´ | 5´ CAC ATT AGG CGC AAT CCA GGT 3´ | 192 bp | 60°C |
| *CXCL3* | 5´ GGG AGC ACC AAC TGA C 3´ | 5´ GAA CCC TCG TAA GAA ATA GTC 3´ | 168 bp | 56°C |
| *CXCL6* | 5´GTC TGG ACC CGG AAG C 3´ | 5´CCC CAC ACT CTT CAA AGT GG3´ | 219 bp | 60°C |
| *CXCL8* | 5´ GTG CAG AGG GTT GTG GA 3´ | 5´ ACC AGG AAT CTT GTA TTG CAT 3´ | 179 bp | 56°C |
| *CXCL10* | 5´ GGC CAT CAA GAA TTT ACT GA 3´ | 5´ CAT TAT AGT GCC AGG GTA GAG 3´ | 292 bp | 54°C |
| *CXCL11* | 5´ AGT GAA AGT GGC AGA TAT TG 3´ | 5´CTT TTC CAG GAC TTC ATA TGT 3´ | 200 bp | 56°C |
| *RPLP0* | 5´ CCT CAT ATC CGG GGG AAT GTG 3´ | 5´ GCA GCA GCT GGC ACC TTA TTG 3´ | 95 bp | 58°C |
| *RPS18* | 5´ CGA TGG GCG GCG GAA AA 3´ | 5´ CAG TCG CTC CAG GTC TTC ACG G 3´ | 283 bp | 58°C |

**Supplementary Table S2.** Summary of all analyses performed.

| Chemokine | [1]Cervical Cancer-derived cell lines (RNAseq) | | | [1]HaCaT-transduced cell models (RNAseq) | | [2]HaCaT-transduced cell models (2-ΔΔCp) | | [1]Expression profile of CESC biopsies from TCGA (RNAseq) | [3]Overall Survival Analysis (upregulation / prognosis) |
|---|---|---|---|---|---|---|---|---|---|
| | SiHa | HeLa | C33A | E6/E7 HPV16 | E6/E7 HPV18 | E6/E7 HPV16 | E6/E7 HPV16 | | |
| *CCL2* | ▼ -5.13 | ▼ -3.93 | ▼ -11.03 | ▲ 4.62 | ▲ 3.94 | ▲ 2.95 | ▲ 3.03 | NS | NS |
| *CCL28* | ▲ 1.66 | ▼ -4.99 | ▼ -5.54 | ▲ 1.48 | ▲ 4.28 | ▲ 2.45 | ▲ 6.94 | ▲ 0.69 | NS |
| *CXCL1* | ▼ -5.32 | ▼ -9.73 | ▼ -8.24 | ▲ 3.54 | ▲ 3.62 | ▼ -0.18 | ▲ 1.81 | ▲ 4.84 | Worse |
| *CXCL2* | ▲ 5.14 | ▲ 5.30 | NS | ▲ 2.73 | ▲ 4.06 | ▲ 3.82 | ▲ 3.64 | NS | Worse |
| *CXCL3* | ▲ 3.13 | ▲ 3.43 | NS | NS | ▲ 4.75 | ▲ 4.01 | ▲ 6.19 | ▲ 2.41 | Worse |
| *CXCL6* | ▼ -8.09 | ▼ -7.89 | ▼ -8.85 | ▲ 3.02 | ▲ 2.30 | ▲ 4.07 | ▲ 3.26 | ▲ 2.14 | Worse |
| *CXCL8* | ▼ -3.21 | ▼ -2.29 | ▼ -9.66 | ▲ 1.18 | ▲ 1.74 | ▲ 0.85 | ▲ 3.01 | ▲ 5.22 | Worse |
| *CXCL10* | ▲ 3.62 | ▲ 5.05 | NS | ▲ 4.72 | ▲ 6.42 | ▲ 3.99 | ▲ 5.58 | ▲ 5.72 | Better |
| *CXCL11* | ▲ 4.36 | ▲ 3.82 | NS | NS | ▲ 2.55 | ▲ 2.42 | ▲ 2.50 | ▲ 3.94 | Worse |

* The table summarizes the results of each analysis executed in the present study. [1]The Differential Expression Analysis was performed on Cervical Cancer-derived cell lines, HaCaT-transduced cell models, and CESC biopsies from the TCGA Database. [2]For the Relative Expression Analysis on HaCaT-transduced cell models, to normalize the expression data, the *RPS18* and *RPLP0* were taken as reference genes. The data displayed are the means of the two replicates of each reference gene. [3]Overall survival data correlates the upregulation of the chemokine panel with the patient´s prognosis. All the resulting outputs from the Differential Expression Analysis (RNAseq) and the Relative Expression (2-ΔΔCp) are shown as Log2 Fold-Change. ▲ Upregulation; ▼ Downregulation; (NS) Non-significant.
